# Supplementary figures and images for: Bacterial and host enzymes modulate the pro-inflammatory response elicited by the peptidoglycan of Lyme disease agent Borrelia burgdorferi
Source: PLoS Pathog. 2025 Jul 7;21(7):e1013324. doi: 10.1371/journal.ppat.1013324 (PMC12279116; doi:10.1371/journal.ppat.1013324)

**A**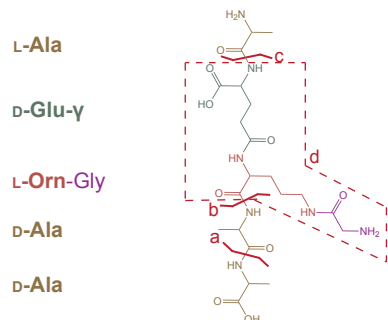

L-Ala-D-Glu-L-Orn(Gly)-D-Ala-D-Ala

**B**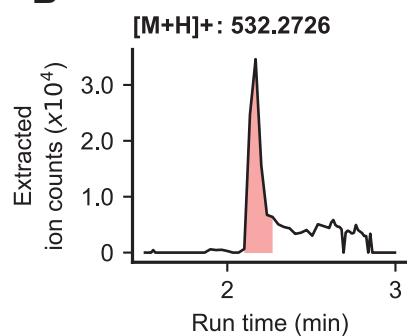**C**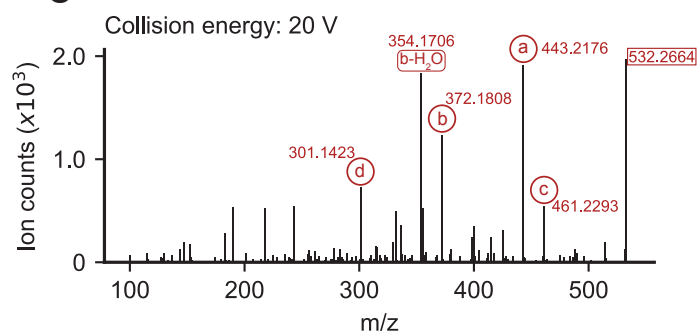

**Figure S1**

Supplement: S1 Fig — Schematic outlining each amino acid in l-Ala-d-Glu-l-Orn(Gly)-d-Ala-d-Ala, along with notations of the origin of each identified fragment after MS/MS. B. EIC profile of the identified [M+H]+ profile. The peak of interest is shaded in red. C. The MS2 spectrum at the run time where the EIC peak in (B) is at maximum intensity. The collision energy used to fragment this molecule is indicated, and each identified fragment is marked with a letter corresponding to the schematic in (A). (PDF) [file ppat.1013324.s001.pdf]

**A**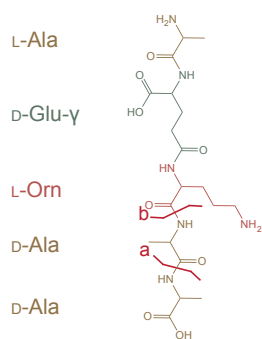

L-Ala-D-Glu-L-Orn-D-Ala-D-Ala

**B**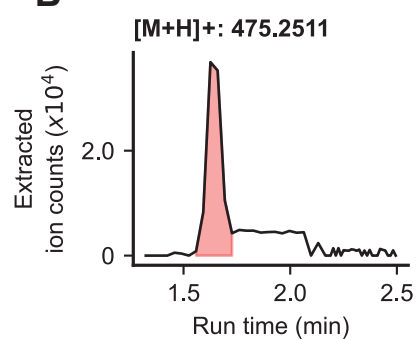**C**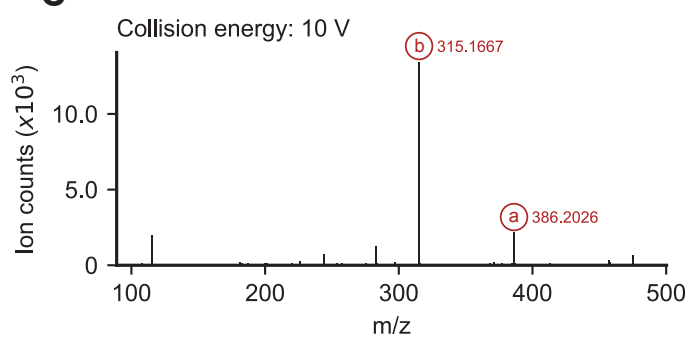

**Figure S2**

Supplement: S2 Fig — Schematic outlining each amino acid in l-Ala-d-Glu-l-Orn-d-Ala-d-Ala, along with notations of the origin of each identified fragment after MS/MS. B. EIC profile of the identified [M+H]+ profile. The peak of interest is shaded in red. C. The MS2 spectrum at the run time where the EIC peak in (B) is at maximum intensity. The collision energy used to fragment this molecule is indicated, and each identified fragment is marked with a letter corresponding to the schematic in (A). (PDF) [file ppat.1013324.s002.pdf]

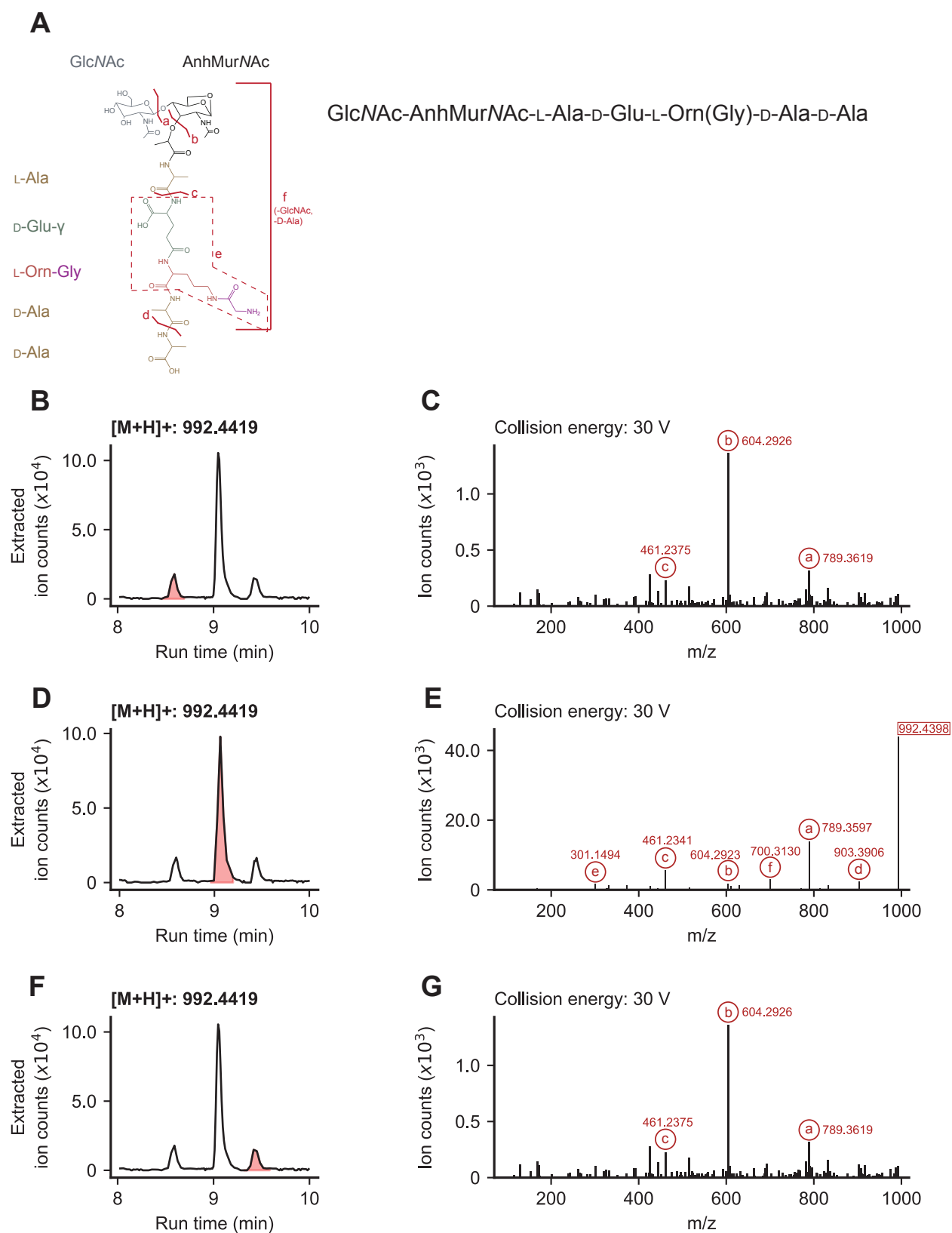

**Figure S3**

Supplement: S3 Fig — Schematic outlining the sugars and amino acids in GlcNAc-AnhMurNAc-l-Ala-d-Glu-l-Orn(Gly)-d-Ala-d-Ala, along with notations of the origin of each identified fragment after MS/MS. B. EIC profile of the identified [M+H]+ profile of peak 1, shaded in red. C. The MS2 spectrum at the run time where EIC peak 1 in (B) is at maximum intensity. D. EIC profile of the identified [M+H]+ profile of peak 2, shaded in red. E. The MS2 spectrum at the run time where EIC peak 2 in (D) is at maximum intensity. F. EIC profile of the identified [M+H]+ profile of peak 3, shaded in red. G. The MS2 spectrum at the run time where EIC peak 3 in (F) is at maximum intensity. For (C), (E), and (G), the collision energies used to fragment this molecule are noted in the titles, and each identified fragment is marked with a letter corresponding to the schematic in (A). (PDF) [file ppat.1013324.s003.pdf]

**A**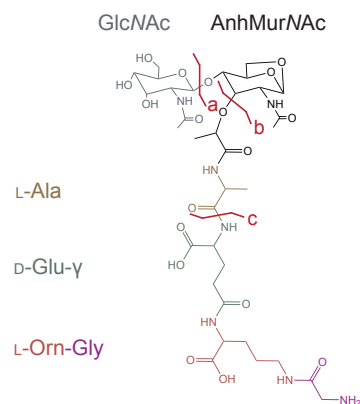

GlcNAc-AnhMurNAc-L-Ala-D-Glu-L-Orn(Gly)

**B**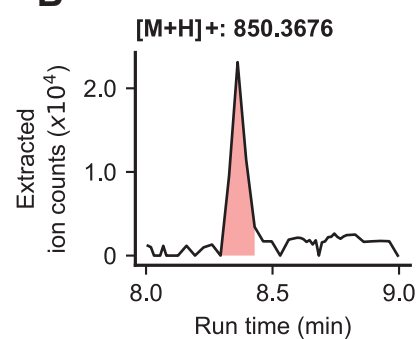**C**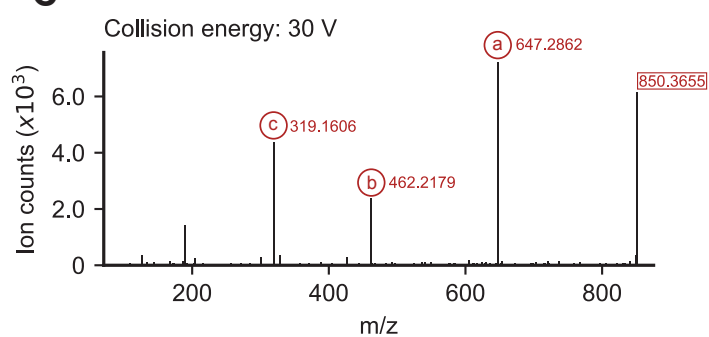

**Figure S4**

Supplement: S4 Fig — A. Schematic outlining the sugars and amino acids in GlcNAc-AnhMurNAc-l-Ala-d-Glu-l-Orn(Gly), along with notations of the origin of each identified fragment after MS/MS. B. EIC profile of the identified [M+H]+ profile. The peak of interest is shaded in red. C. The MS2 spectrum at the run time where the EIC peak in (B) is at maximum intensity. The collision energy used to fragment this molecule is indicated, and each identified fragment is marked with a letter corresponding to the schematic in (A). (PDF) [file ppat.1013324.s004.pdf]

**A**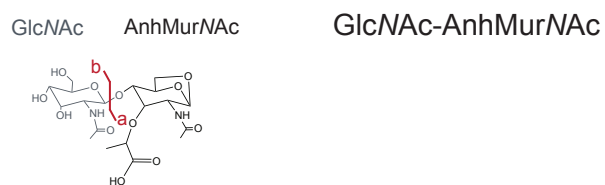**B**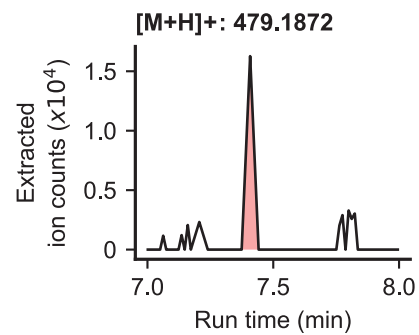**C**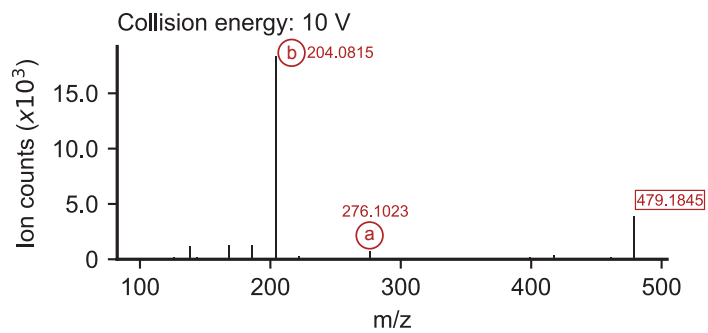**Figure S5**

Supplement: S5 Fig — Schematic outlining each sugar in GlcNAc-AnhMurNAc, along with notations of the origin of each identified fragment after MS/MS. B. EIC profile of the identified [M+H]+ profile. The peak of interest is shaded in red. C. The MS2 spectrum at the run time where the EIC peak in (B) is at maximum intensity. The collision energy used to fragment this molecule is indicated, and each identified fragment is marked with a letter corresponding to the schematic in (A). (PDF) [file ppat.1013324.s005.pdf]

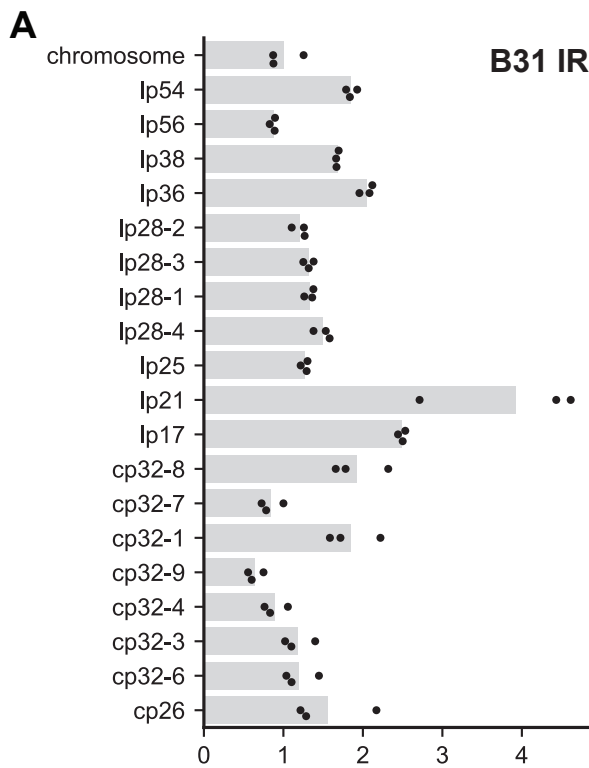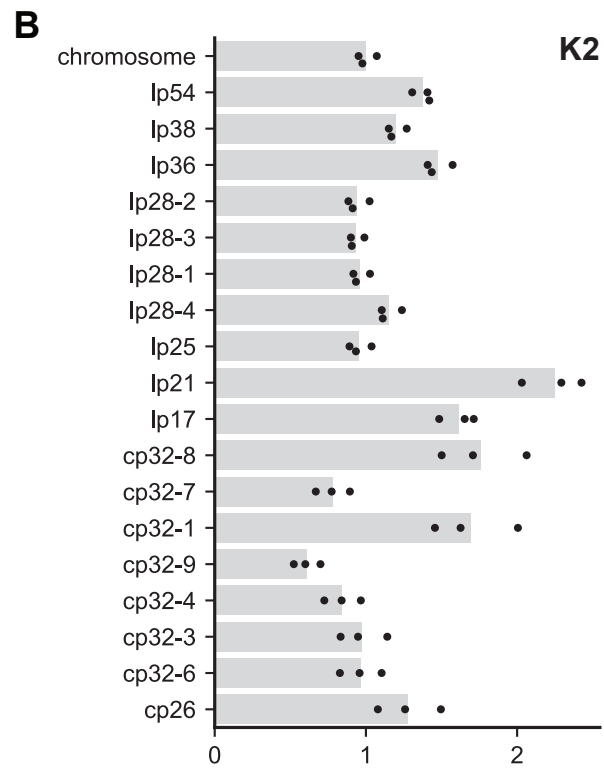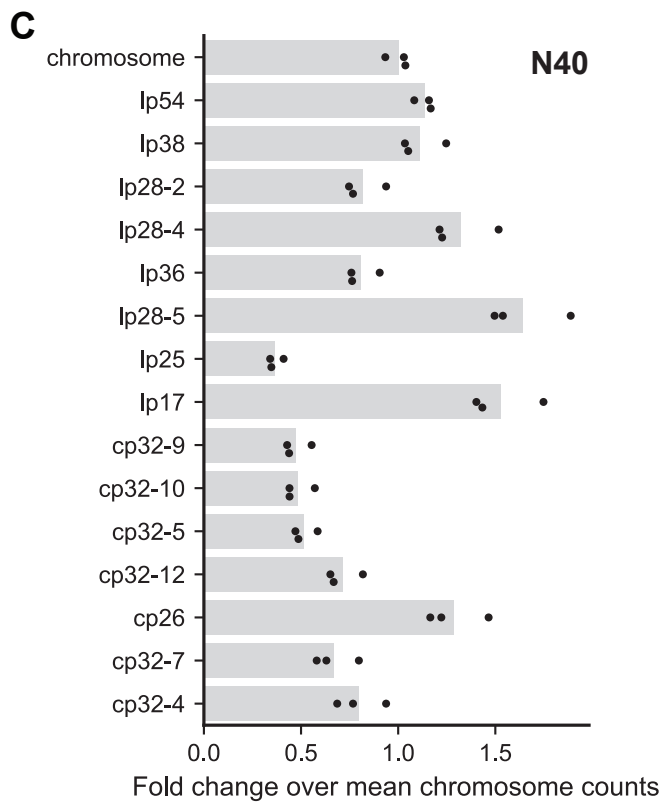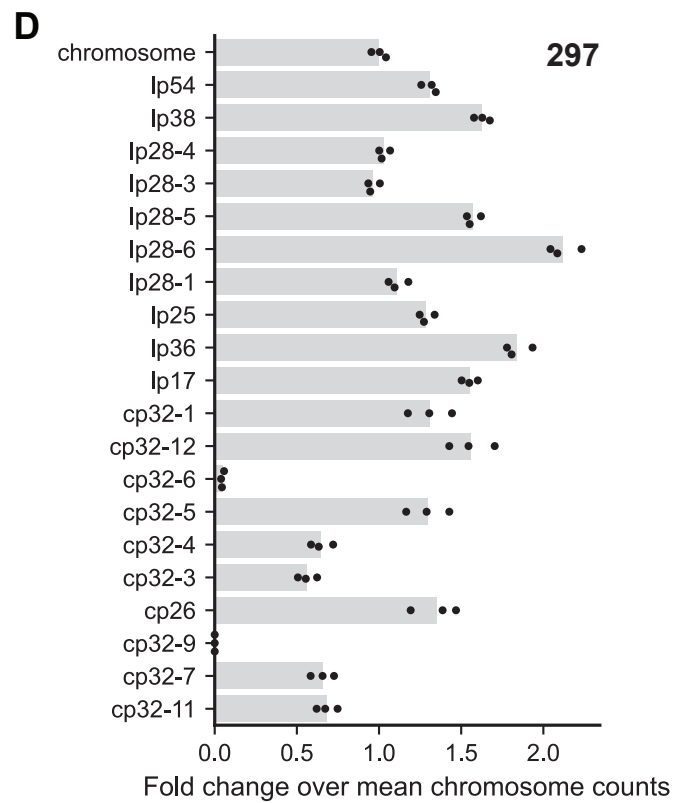

**Figure S6**

Supplement: S6 Fig — Fold change over mean chromosome read counts for each plasmid present in strains: A. B31 IR, B. K2, C. N40, and D. 297. For each strain, a fold change near 0 (< 0.25) indicates that it is missing that plasmid. (PDF) [file ppat.1013324.s006.pdf]

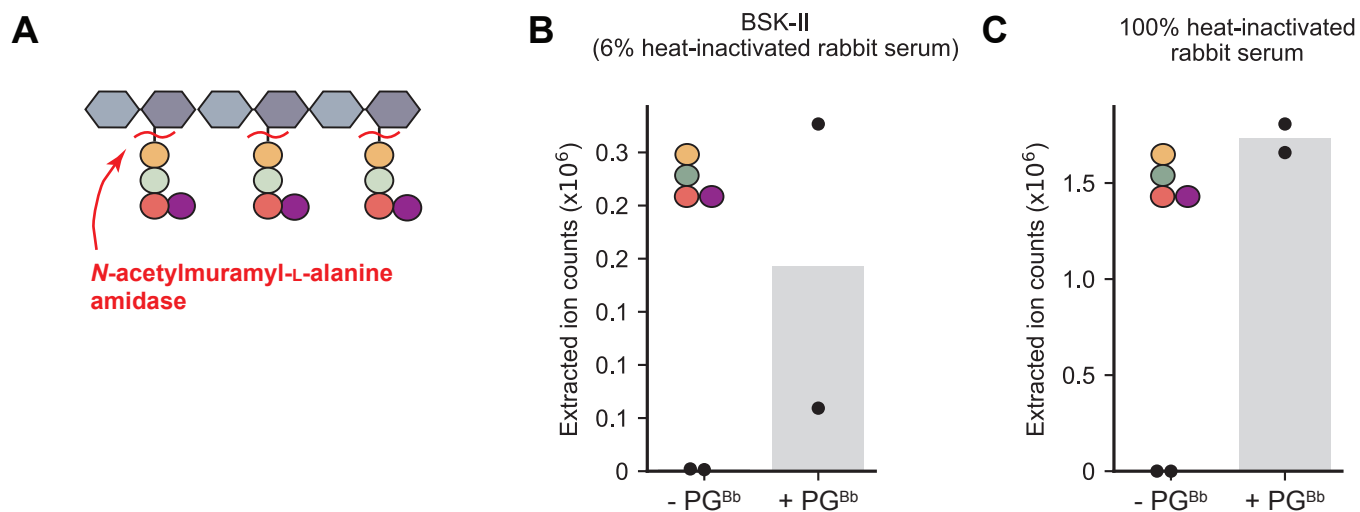

**Figure S7**

Supplement: S7 Fig — BSK-II or heat-inactivated rabbit serum was incubated at 37ºC in the presence or absence of purified PGBb sacculi for 24 h prior to LC-MS. A. Schematic of N-acetylmuramyl-l-alanine amidase activity when mixed with purified PGBb. Cut sites by a N-acetylmuramyl-l-alanine amidase are shown by red curvy lines. B. Digestion in BSK-II. C. Digestion in heat-inactivated rabbit serum (100%). For B-C, plots show the extracted ion count (EIC) for the l-Ala-d-Glu-l-Orn(Gly), the predominant expected digestion product of PGBb sacculi by a N-acetylmuramyl-l-alanine amidase. For both panels, the bar shows the mean and the dots represent the data of two biological replicates of BSK-II media and rabbit sera sourced from different lot numbers. (PDF) [file ppat.1013324.s007.pdf]

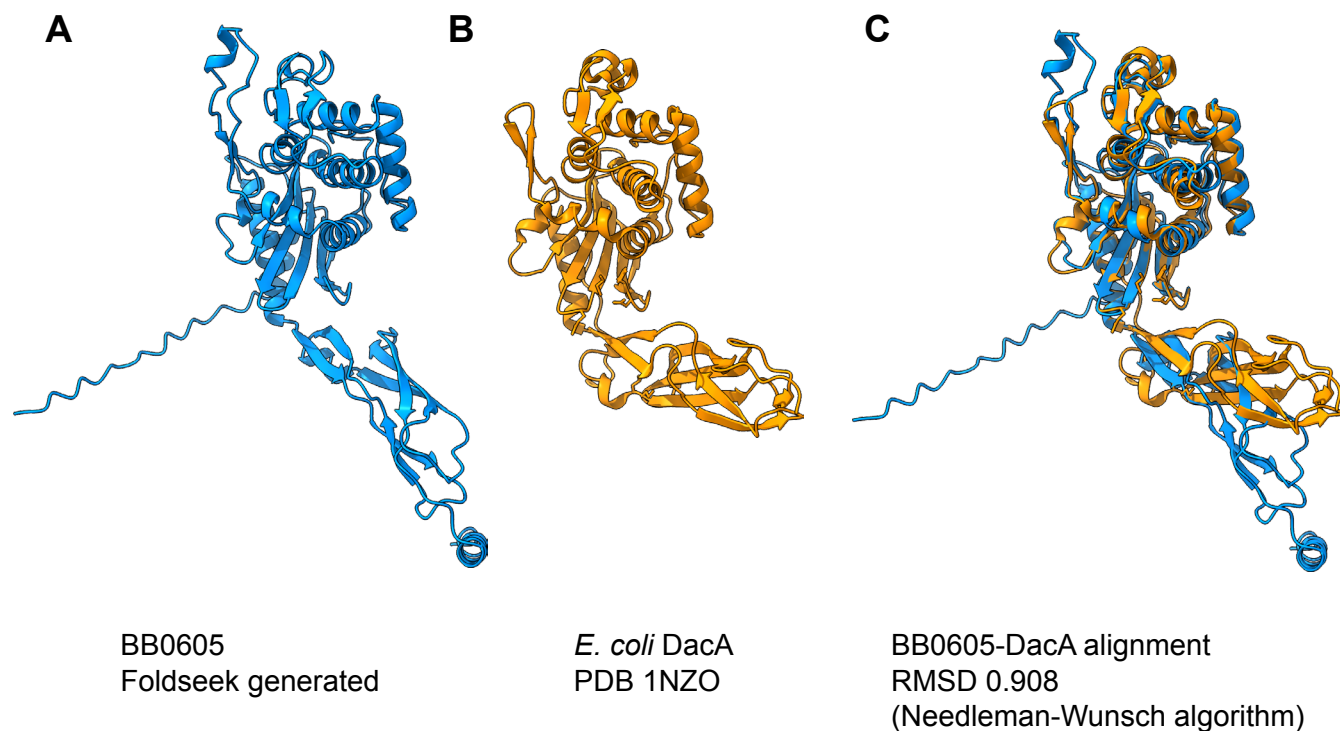

**Figure S8**

Supplement: S8 Fig — Predicted structure of B. burgdorferi BB0605 using Foldseek [109]. B. Structure of E. coli DacA obtained from the Protein Data Bank (PDB) [110,111]. C. Alignment of BB0605 and DacA, performed using ChimeraX [112]. The root mean square deviation (RMSD) from alignment is presented, along with the alignment algorithm used. (PDF) [file ppat.1013324.s008.pdf]

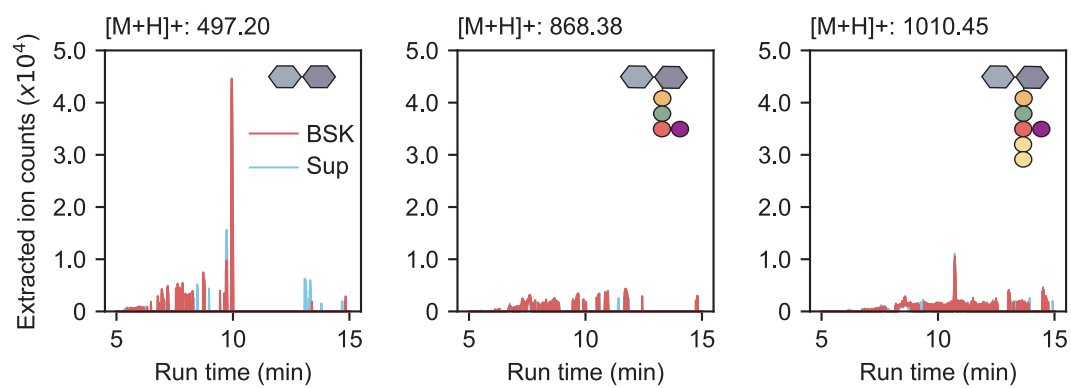

**Figure S9**

Supplement: S9 Fig — Plots showing the extracted ion counts of GlcNAc-MurNAc, GlcNAc-MurNAc-l-Ala-d-Glu-l-Orn(Gly), and GlcNAc-MurNAc-l-Ala-d-Glu-l-Orn(Gly)-d-Ala-d-Ala in culture supernatants (Sup) compared to medium alone (BSK). (PDF) [file ppat.1013324.s009.pdf]

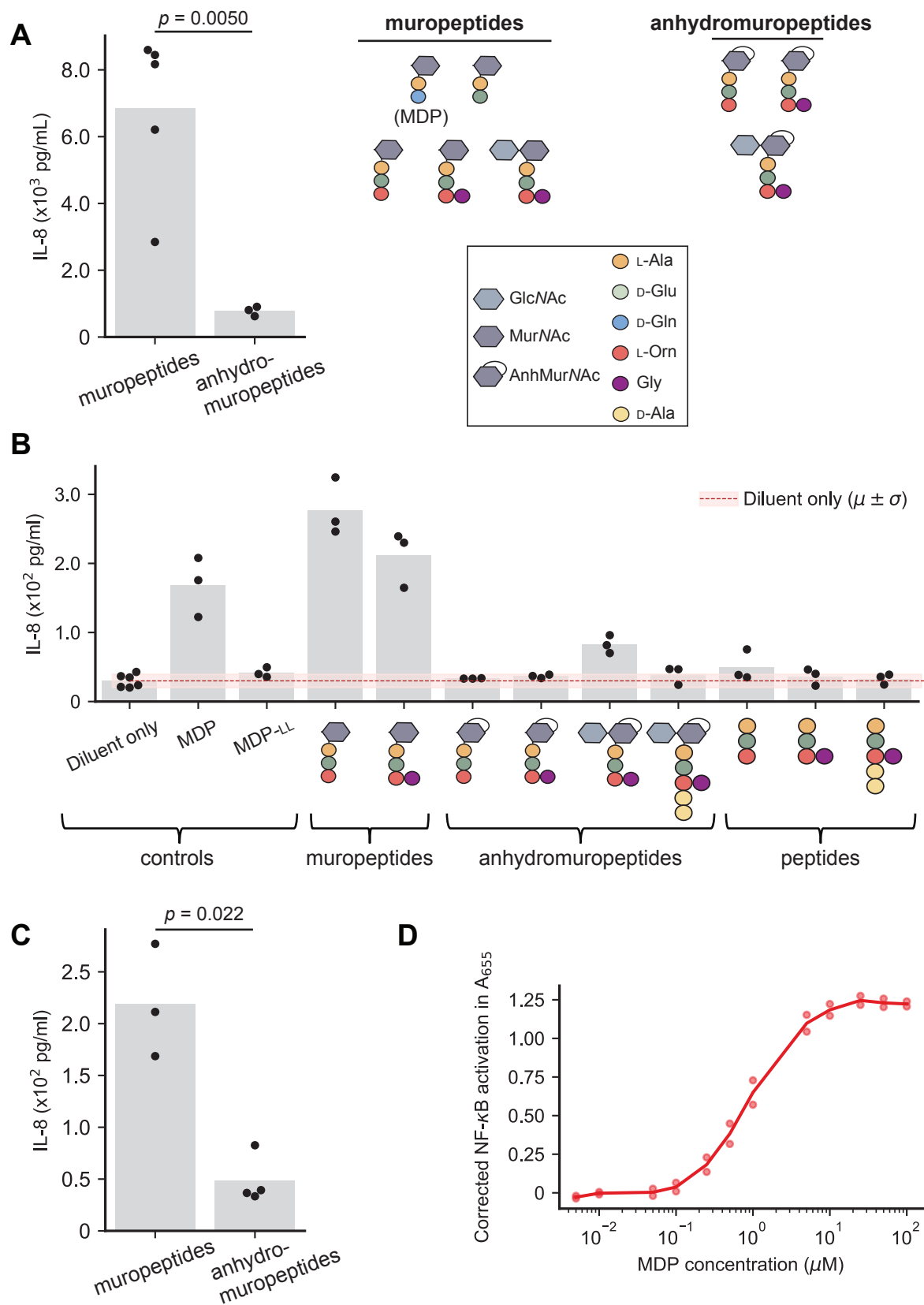

**Figure S11**

Supplement: S11 Fig — A. Comparison between MurNAc-containing and AnhMurNAc-containing species in Fig 5A. The schematic of the PGBb species in each group is shown, along with a legend that defines each chemical moiety. Dots represent the means of each PGBb compound. Bar heights represent the means for each compound group. The groups were compared using a Welch’s t-test to account for different standard deviations and N values. B. Plot showing IL-8 production in differentiated THP-1 cells in the presence of the indicated PGBb fragments. The error bars represent standard deviation of the mean, and bar height represents the mean. Dots represent data from three biological replicates. C. Comparison of MurNAc-containing and AnhMurNAc-containing species in (B), including MDP as in (A). Dots represent the means of each PGBb compound. Bar heights represent the means for each compound group. The groups were compared using a Welch’s t-test to account for different standard deviations and N values. D. Dose-response curve of hNOD2 reporter cells to MDP in MilliQ H2O. The line connects the mean measurements of each concentration, and the dots represent technical replicates. (PDF) [file ppat.1013324.s011.pdf]

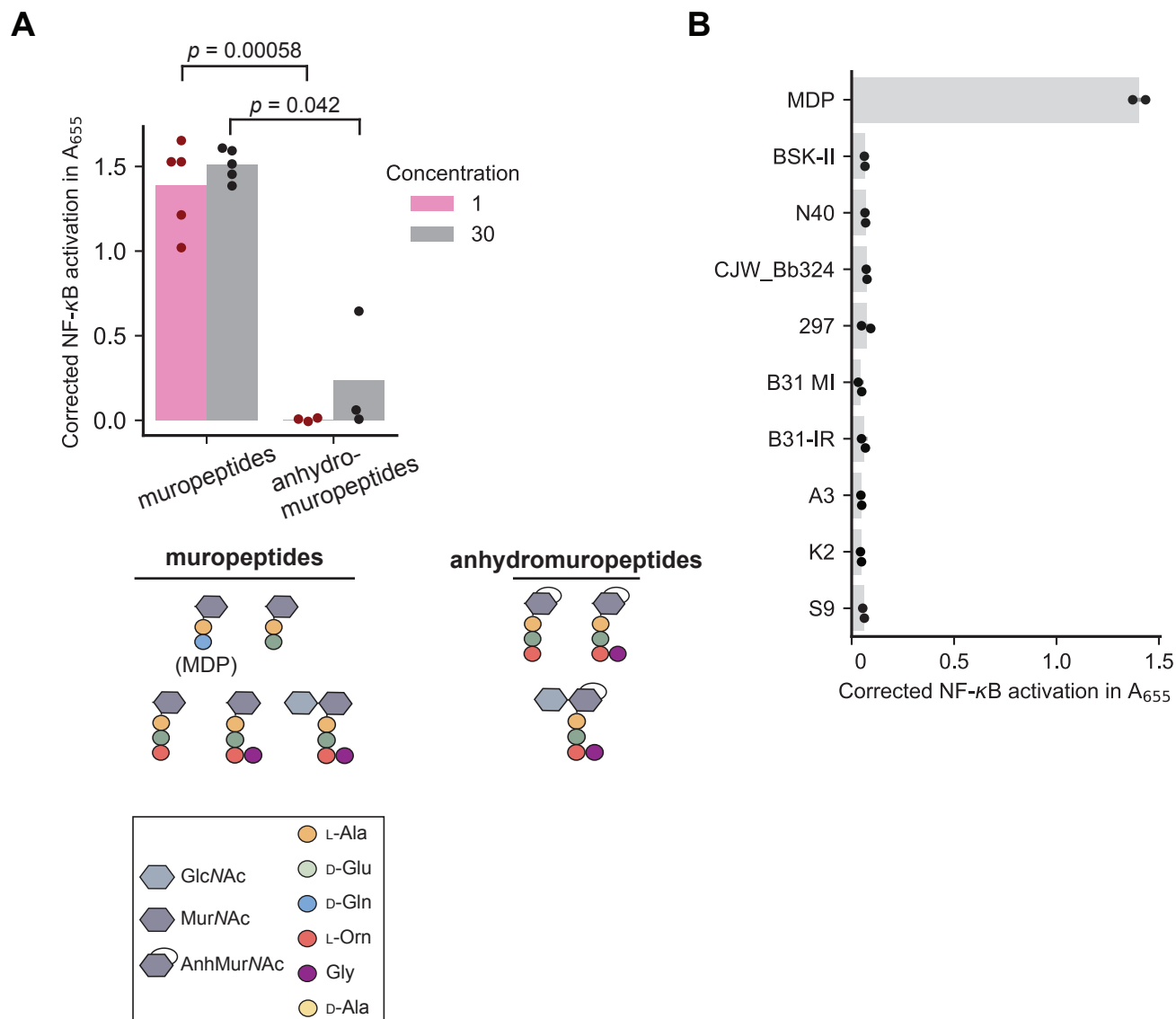

**Figure S12**

Supplement: S12 Fig — A. NOD2 data from Fig 5B with PGBb grouped based on the presence of a MurNAc or AnhMurNAc, as shown by the schematics and the legend. Dots represent the means of each PGBb compound. Bar heights represent the means for each compound group. The lines above the plot show pairwise comparisons between each group using Welch’s t-tests to account for different standard deviations and N values. The resulting p-values were adjusted using a Bonferonni correction for multiple comparisons. B. Plot showing the SEAP activity of hNOD2 reporter cells (used after fifth passage) following 16-h exposure to 1 µM MDP (positive control) compared to complete BSK-II medium (negative control), or supernatants of cultures in stationary phase for three days. Each dot is a technical replicate, and the height of each bar represents the mean. (PDF) [file ppat.1013324.s012.pdf]

**A**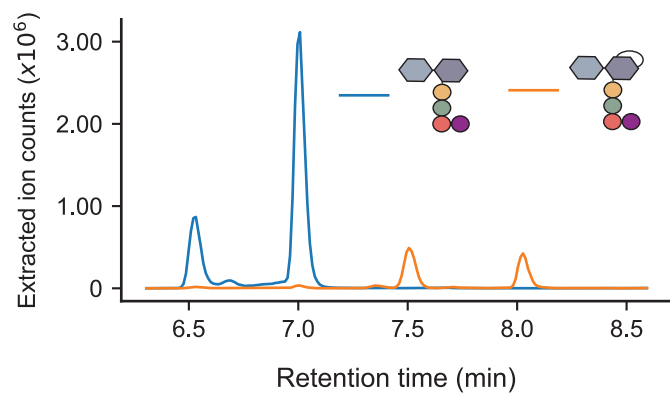**B**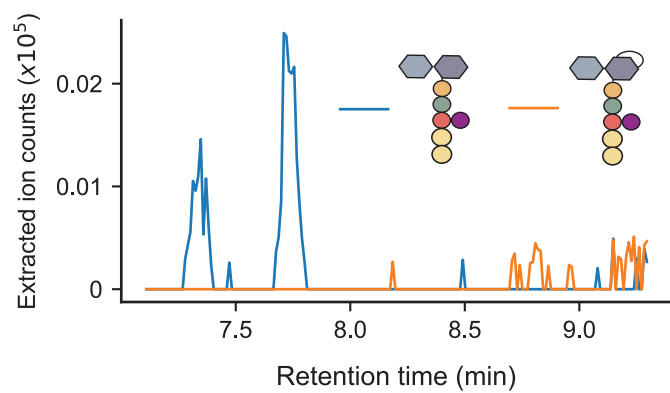**Figure S13**

Supplement: S13 Fig — PGBb sacculi were isolated from 10-day-old stationary phase cultures of B31 IR cells and digested with mutanolysin. Digest products were then analyzed by LC-MS. A. Extracted ion count profiles for GlcNAc-MurNAc-l-Ala-d-Glu-l-Orn(Gly) (blue) vs. GlcNAc-AnhMurNAc-l-Ala-d-Glu-l-Orn(Gly) (orange). B. Extracted ion count profiles for GlcNAc-MurNAc-l-Ala-d-Glu-l-Orn(Gly)-d-Ala-d-Ala (blue) vs. GlcNAc-AnhMurNAc-l-Ala-d-Glu-l-Orn(Gly)-d-Ala-d-Ala (orange). (PDF) [file ppat.1013324.s013.pdf]

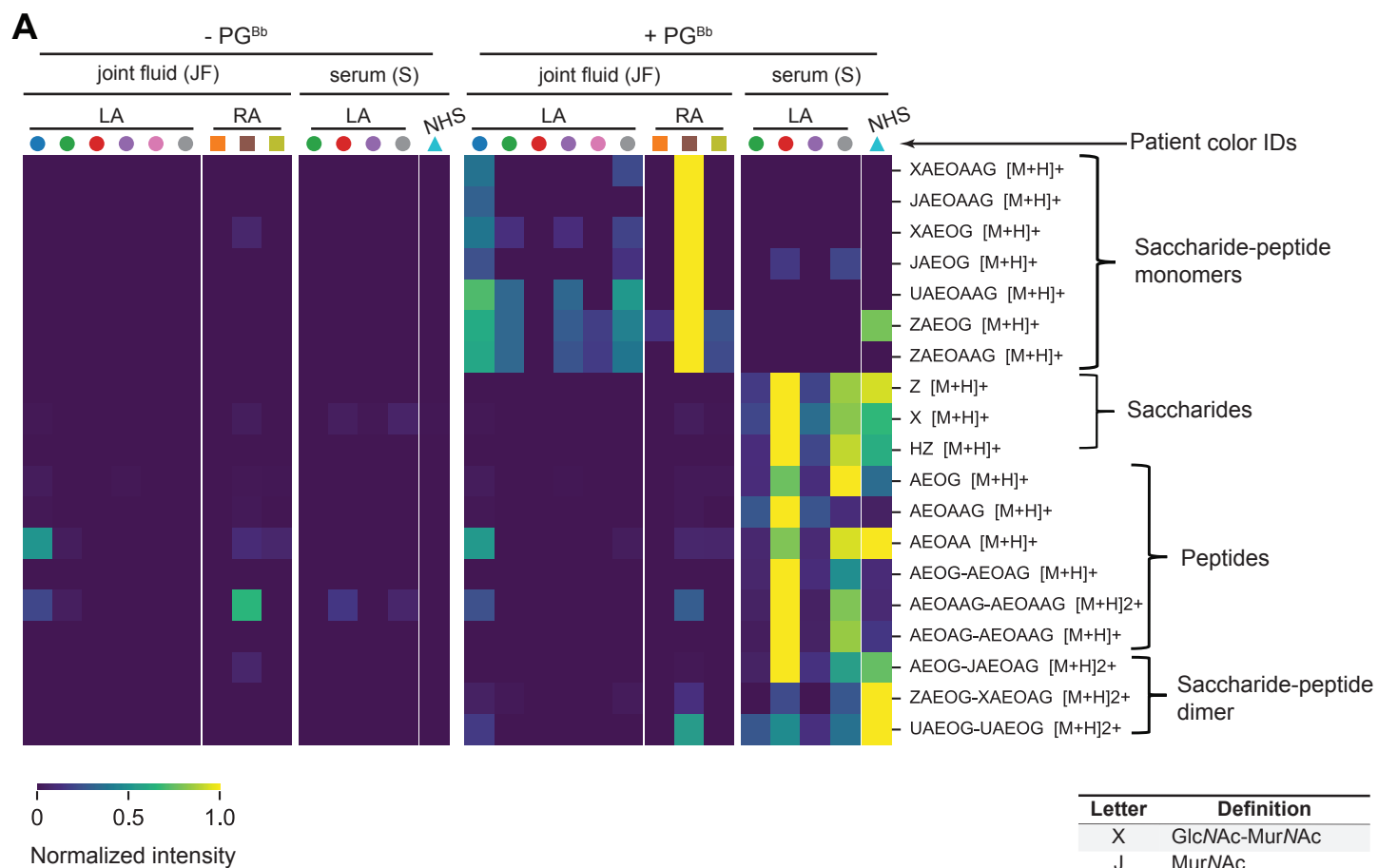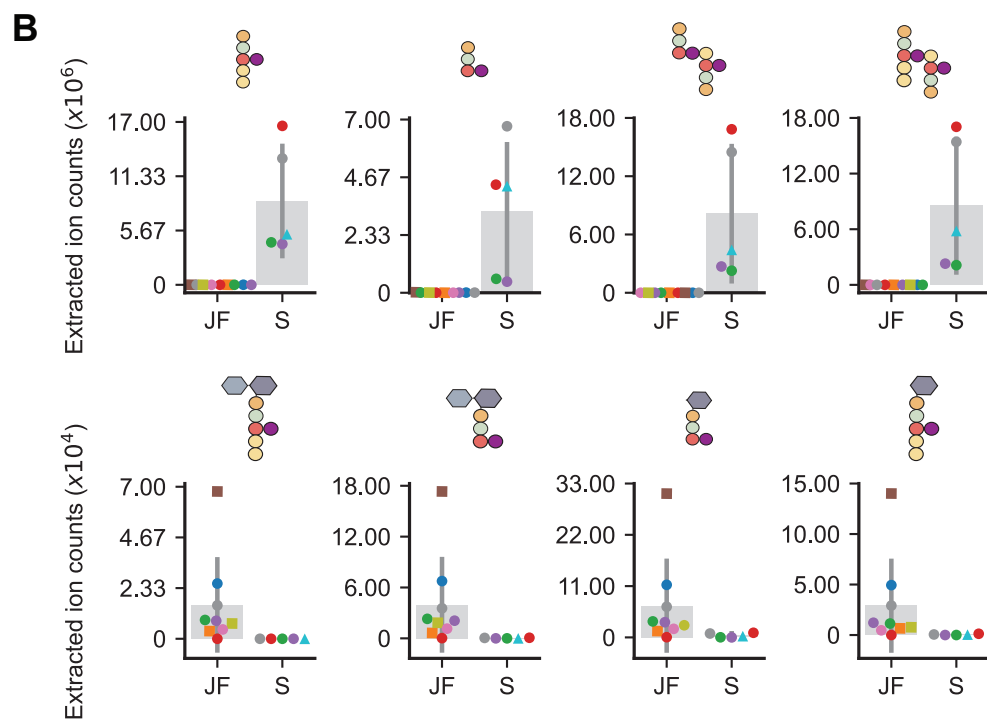

**Figure S14**

Supplement: S14 Fig — This heatmap is similar to Fig 6A except that negative control are samples in which no sacculi were added. Samples were incubated with MilliQ H2O (- PGBb sacculi) or with PGBb sacculi (+ PGBb sacculi) for 6 h, then the resulting reaction products were analyzed by LC-MS. PGBb species were detected by their predicted [M+H] value (S1 Dataset). All samples were derived from Lyme arthritis patients whose identities (IDs) are color-coded as in Fig 6A. The accompanying table contains the key to interpret the PGBb fragment species in the heatmap. B. Representative extracted ion counts for peptide or sugar-peptide conjugate digestion products in serum and joint fluid samples predicted based on their masses. Each dot was derived from integrating the relevant EIC peak. These are the same fragments and analysis as in Fig 6B, but with a linear scale on the y-axis. (PDF) [file ppat.1013324.s014.pdf]
