# Supplementary material for: Bacterial and host enzymes modulate the pro-inflammatory response elicited by the peptidoglycan of Lyme disease agent Borrelia burgdorferi
Source: PLoS Pathog. 2025 Jul 7;21(7):e1013324. doi: 10.1371/journal.ppat.1013324 (PMC12279116; doi:10.1371/journal.ppat.1013324)
Supplement: S10 Fig — A. Predicted structure of BB0259 using Foldseek [109]. B. Structure of E. coli Slt70 obtained from the Protein Data Bank (PDB) [111,113]. C. Alignment of BB0259 and Slt70 using ChimeraX [112]. The root mean square deviation from alignment is presented, along with the alignment algorithm used. (PDF) [file ppat.1013324.s010.pdf]

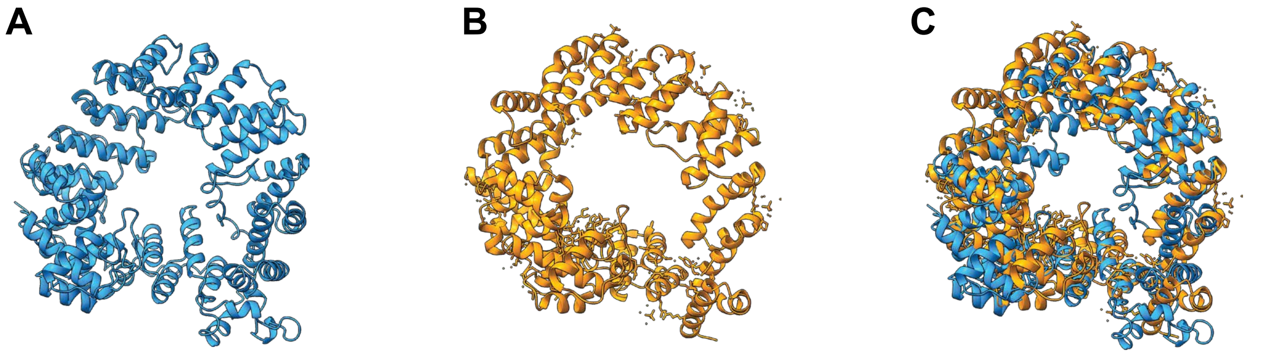

BB0259
Foldseek generated

E. coli Slt70
PDB 1QSA

BB0259-Slt70 alignment
RMSD 1.302
(Needleman-Wunsch algorithm)

Figure S10
