# Supplementary material for: Bacterial and host enzymes modulate the pro-inflammatory response elicited by the peptidoglycan of Lyme disease agent Borrelia burgdorferi
Source: PLoS Pathog. 2025 Jul 7;21(7):e1013324. doi: 10.1371/journal.ppat.1013324 (PMC12279116; doi:10.1371/journal.ppat.1013324)
Supplement: S1 Table — The letter key refers to the heatmap table in Fig 6A. The peptidoglycan fragment masses were generated by summing the masses of each individual subunit and subtracting the mass of water with each addition. (PDF) [file ppat.1013324.s015.pdf]

№	PG fragment candidate	Letter key	Calculated mass	Detected mass	Representative image
1	L-Ala-D-Glu-L-Orn(Gly)-D-Ala-D-Ala	AEOAAG	531.2652745	532.2725505 [M+H]+	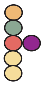
2	L-Ala-D-Glu-L-Orn-D-Ala-D-Ala	AEOAA	474.2438112	475.2510872 [M+H]+	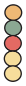
3	Glc*N*Ac-AnhMur*N*Ac-L-Ala-D-Glu-L-Orn(Gly)-D-Ala-D-Ala	ZAEOAAG	991.4345841	992.4418601 [M+H]+	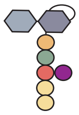
4	Glc*N*Ac-AnhMur*N*Ac-L-Ala-D-Glu-L-Orn(Gly)	ZAEOG	849.3603575	850.3676335 [M+H]+	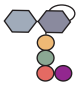
5	Glc*N*Ac-AnhMur*N*Ac	Z	478.1798743	479.1871503 [M+H]+	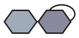
6	L-Ala-D-Glu-L-Orn	AEO	332.1695846	333.1768606 [M+H]+	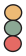
7	L-Ala-D-Glu-L-Orn(Gly)	AEOG	389.1910479	390.1983239 [M+H]+	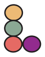
8	GlcNAc-Mur*N*Ac-L-Ala-D-Glu-L-Orn(Gly)	XAEOG	867.3709225	868.3781985 [M+H]+	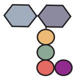
9	Glc*N*Ac-Mur*N*Ac-L-Ala-D-Glu-L-Orn(Gly)-D-Ala-D-Ala	XAEOAAG	1009.445149	1010.452425 [M+H]+	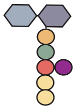
10	Glc*N*Ac-Mur*N*Ac	X	496.1904393	497.1977153 [M+H]+	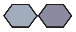
11	Mur*N*Ac-L-Ala-D-Glu	JAE	493.1907736	494.1980496 [M+H]+	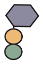
12	Mur*N*Ac-L-Ala-D-Glu-L-Orn	JAEO	607.2700869	608.2773629 [M+H]+	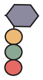
13	Mur*N*Ac-L-Ala-D-Glu-L-Orn(Gly)	JAEOG	664.2915502	665.2988262 [M+H]+	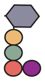
14	AnhMur*N*Ac-L-Ala-D-Glu-L-Orn	UAEO	589.2595219	590.2667979 [M+H]+	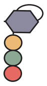
15	AnhMur*N*Ac-L-Ala-D-Glu-L-Orn(Gly)	UAEOG	646.2809852	647.2882612 [M+H]+	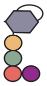
16	L-Ala-D-Glu-L-Orn[Gly—D-Ala-L-Orn(Gly)-D-Glu-L-Ala]	AEOG-AEOAG	831.4086444	832.4159204 [M+H]+ 416.7115982 [M+H]2+	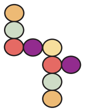

17	L-Ala-D-Glu-L-Orn[Gly—D-Ala-L-Orn(Gly)-D-Glu-L-Ala]-D-Ala-D-Ala	AEOAAG-AEOAG	973.482871	974.490147 [M+H]+ 487.7487115 [M+H]2+	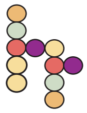
18	Mur*N*Ac-L-Ala-D-Glu-L-Orn(Gly)-D-Ala-D-Ala	JAEOAAG	806.3657768	807.3730528 [M+H]+	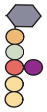
19	AnhMur*N*Ac-L-Ala	UA	346.1376153	347.1448913 [M+H] 174.0760837 [M+H]2+	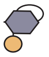
20	AnhMur*N*Ac-L-Ala-D-Glu-L-Orn(Gly)-D-Ala-D-Ala	UAEOAAG	788.3552118	789.3624878 [M+H]+	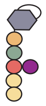
21	Glc*N*Ac-Glc*N*Ac-AnhMur*N*Ac	HZ	681.2592396	682.2665156 [M+H]+	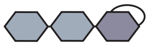
22	L-Ala-D-Glu-L-Orn[Gly—D-Ala-L-Orn(Gly)-D-Glu-L-Ala-Mur*N*Ac]	AEOG-JAEOAG	1106.509147	1107.516423 [M+H]+ 554.2618494 [M+H]2+	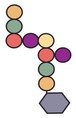
23	Glc*N*Ac-AnhMur*N*Ac-L-Ala-D-Glu-L-Orn[Gly—D-Ala-L-Orn(Gly)-D-Glu-L-Ala-Mur*N*Ac-Glc*N*Ac]	ZAEOG-XAEOAG	1769.757829	1770.765105 [M+H]+ 885.8861903 [M+H]2+	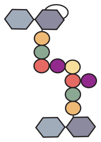

S1 Table. Common PG species referenced within this manuscript. The letter key refers to the heatmap table in Fig 6A. The peptidoglycan fragment masses were generated by summing the masses of each individual subunit and subtracting the mass of water with each addition.
