# Supplementary material for: Bacterial and host enzymes modulate the pro-inflammatory response elicited by the peptidoglycan of Lyme disease agent Borrelia burgdorferi
Source: PLoS Pathog. 2025 Jul 7;21(7):e1013324. doi: 10.1371/journal.ppat.1013324 (PMC12279116; doi:10.1371/journal.ppat.1013324)
Supplement: S2 Table — (PDF) [file ppat.1013324.s016.pdf]

Strain	Relevant genotype/description	Source
B31 MI	Infectious, mouse isolate	[1]
A3	B31 MI *cp9*$^-$	[2]
K2	B31-A3-68 lp25[*bbe02*::P$_{flgB}$-*aphI*] lp56$^-$ lp5$^-$	[3]
S9	B31-A3-68 lp25[*bbe02*::P$_{flgB}$-*aphI*] lp56$^-$ lp5$^-$	[3]
N40	Infectious, tick isolate	[4]
N40 D10/E9	Clonal isolate of N40	[5]
CJW_Bb324	N40 D10/E9 lp25[*bbe02*::*Bbluc*-*aadA*]	[6]
297	Infectious, patient isolate	[7]
5A18NP1	B31, clone 5A18NP1	[8]
T08TC493	B31, clone 5A18NP1, Chr[*bb0605*::Himar*1*]	[9]
B31-A	Wild type, noninfectious, high passage	[10]
Δ*bb0531*	B31-A Chr[*bb0531*::P$_{flgB}$-*aphI*]	[10]
Δ*bb0259*	B31-A Chr[*bb0259::*P$_{flgB}$-*aphI*]	[10]
bb0259^{com}	Bb612//pBSV2G-P$_{flgB}$-*bb0259*	[10]
B31 MI IR, (B31 IR)	B31 MI, clonal isolate	[11]

S2 Table. *B. burgdorferi* strains used in this study.

References

1. Fraser CM, Casjens S, Huang WM, Sutton GG, Clayton R, Lathigra R, et al. Genomic sequence of a Lyme disease spirochaete, *Borrelia burgdorferi*. Nature. 1997;390(6660):580-6. doi: 10.1038/37551. PubMed PMID: 9403685.
2. Elias AF, Stewart PE, Grimm D, Caimano MJ, Eggers CH, Tilly K, et al. Clonal Polymorphism of Borrelia burgdorferi Strain B31 MI: Implications for Mutagenesis in an Infectious Strain Background. Infection and Immunity. 2002;70(4):2139-50. doi: doi:10.1128/IAI.70.4.2139-2150.2002. PubMed PMID: 11895980.
3. Rego ROM, Bestor A, Rosa PA. Defining the Plasmid-Borne Restriction-Modification Systems of the Lyme Disease Spirochete *Borrelia burgdorferi*. Journal of Bacteriology. 2011;193(5):1161-71. doi: doi:10.1128/JB.01176-10. PubMed PMID: 21193609.
4. Barthold SW, Moody KD, Terwilliger GA, Duray PH, Jacoby RO, Steere AC. Experimental Lyme Arthritis in Rats Infected with *Borrelia burgdorferi*. The Journal of Infectious Diseases. 1988;157(4):842-5. doi: 10.1093/infdis/157.4.842. PubMed PMID: 3258003.
5. Leong JM, de Vargas LM, Isberg RR. Binding of cultured mammalian cells to immobilized bacteria. Infection and Immunity. 1992;60(2):683-6. PubMed PMID: 1730504.
6. Chan K, Alter L, Barthold SW, Parveen N. Disruption of *bbe02* by insertion of a luciferase gene increases transformation efficiency of *Borrelia burgdorferi* and allows live imaging in lyme disease susceptible C3H mice. PLoS One. 2015;10(6):e0129532. PubMed PMID: 26069970.
7. Steere AC, Grodzicki RL, Kornblatt AN, Craft JE, Barbour AG, Burgdorfer W, et al. The Spirochetal Etiology of Lyme Disease. New England Journal of Medicine. 1983;308(13):733-40. doi: 10.1056/nejm198303313081301. PubMed PMID: 6828118.

8. Kawabata H, Norris SJ, Watanabe H. BBE02 Disruption Mutants of *Borrelia burgdorferi* B31 Have a Highly Transformable, Infectious Phenotype. Infection and Immunity. 2004;72(12):7147-54. doi: doi:10.1128/IAI.72.12.7147-7154.2004. PubMed PMID: 15557639.
9. Lin T, Gao L, Zhang C, Odeh E, Jacobs MB, Coutte L, et al. Analysis of an Ordered, Comprehensive STM Mutant Library in Infectious *Borrelia burgdorferi*: Insights into the Genes Required for Mouse Infectivity. PLOS ONE. 2012;7(10):e47532. doi: 10.1371/journal.pone.0047532. PubMed PMID: 23133514.
10. Xu H, Hu B, Flesher DA, Liu J, Motaleb MA. BB0259 Encompasses a Peptidoglycan Lytic Enzyme Function for Proper Assembly of Periplasmic Flagella in *Borrelia burgdorferi*. Frontiers in Microbiology. 2021;12. doi: 10.3389/fmicb.2021.692707. PubMed PMID: 34659138.
11. Takacs CN, Wachter J, Xiang Y, Ren Z, Karaboja X, Scott M, et al. Polyploidy, regular patterning of genome copies, and unusual control of DNA partitioning in the Lyme disease spirochete. Nature Communications. 2022;13(1):7173. doi: 10.1038/s41467-022-34876-4. PubMed PMID: 36450725.
