# Supplementary material for: Bacterial and host enzymes modulate the pro-inflammatory response elicited by the peptidoglycan of Lyme disease agent Borrelia burgdorferi
Source: PLoS Pathog. 2025 Jul 7;21(7):e1013324. doi: 10.1371/journal.ppat.1013324 (PMC12279116; doi:10.1371/journal.ppat.1013324)
Supplement: S3 Table — The LC gradient for LC-MS experiments. Buffers A and B are noted in Materials and Methods. (PDF) [file ppat.1013324.s017.pdf]

Time (min)	A%	B%	Flow (mL/min)
0	100	0	0.2
3	100	0	0.2
7	90	10	0.2
15	80	20	0.2
17	5	95	0.2
17.5	5	95	0.4
19.5	5	95	0.4
20.5	100	0	0.4
24	100	0	0.4
24.5	100	0	0.2
26	100	0	0.2

S3 Table. **The LC gradient for LC-MS experiments**. Buffers A and B are noted in Materials and Methods.
