# Supplementary material for: Bacterial and host enzymes modulate the pro-inflammatory response elicited by the peptidoglycan of Lyme disease agent Borrelia burgdorferi
Source: PLoS Pathog. 2025 Jul 7;21(7):e1013324. doi: 10.1371/journal.ppat.1013324 (PMC12279116; doi:10.1371/journal.ppat.1013324)
Supplement: Attachment — Submitted filename: Response-to-reviews.docx [file ppat.1013324.s019.docx]

**Editor’s response**

Thank you for submitting your manuscript to PLOS Pathogens. The study described is very interesting and carefully performed. However, the reviewers had some comments to enhance the manuscript. Therefore, we invite you to submit a revised version of the manuscript that addresses the points raised during the review process.

Authors: Thank you so much for handling our manuscript. We appreciate the time and effort spent on our manuscript. We believe that we have addressed all of the reviewers’ comments; please see our point-by-point response below (in blue). In addition, we have addressed the journal requirements mentioned below.

**Reviewers' Comments:**

Reviewer's Responses to Questions

**Part I - Summary**
Please use this section to discuss strengths/weaknesses of study, novelty/significance, general execution and scholarship.

Reviewer #1: This extremely well executed but complicated study is one of a number from the Jacobs-Wagner group addressing fundamental aspects of Borrelia burgdorferi cell biology, in this case, turnover of peptidoglycan (PG) and release of PG fragments to the extracellular milieu. What makes their work unique is their ability to relate basic biochemical, structural, and genetic analysis of B. burgdorferi PG composition and turnover to the immunopathogenesis of chronic Lyme arthritis –one of the least well understood clinical manifestations of Lyme disease. The starting point is their use of advanced mass spectroscopy approaches to characterize PG fragments B. burgdorferi that accumulate in the culture medium during in vitro growth. They accomplish this using a novel approach – matching mass spec signatures to a computational library of PG monomers and dimers. They then go on to identify two key borrelial enzymes (BB0605, a carboxypeptidase renamed DacA, and BB0259, a lytic transglycosylase renamed MltS) involved in generating the fragments, and they demonstrate that fragments are generated primarily at sites of PG synthesis during spirochete replication. The unexpected finding (at least for this reviewer) is that the sugar containing fragments have an anhydro chemical modification that renders them relatively invisible to human PG innate sensing mechanisms (e.g., NOD2), a result that conflicts with their previously published findings. They address this discrepancy by showing that PG hydrolytic enzymes in synovial fluid can release proinflammatory PG fragments when incubated with PG sacculi, leading them to propose that post-treatment Lyme arthritis results from breakdown of PG sacculi from dead spirochetes acting as immunostimulants/adjuvants to drive autoimmune responses.

Thank you for the excellent summary of our work.

Without question, this work is a technical tour de force that deals with PG synthesis and turnover in B. burgdorferi at an unprecedented depth and with analytic and computational methodologies new to the field. Moreover, it can be argued that the emphasis in the current work on PG fragments derived from dead spirochetes makes sense given that chronic Lyme arthritis occurs following therapy when live organisms (indeed, even DNA detectable by PCR) are no longer present. Moreover, these new results raise interesting new questions about the balance between inflammation and immune evasiveness during B. burgdorferi infection that should stimulate work in the field. On the other hand, the work has two interrelated weaknesses. One is the not entirely convincing explanation for the discrepancy between the culture supernatant experiments here and reported previously. Nevertheless, it should be noted that the authors are to be commended for their forthright acknowledgement of this discordance and their willingness to set the record straight. The other is that it adds to the experimental mishaps that have marred efforts over the years to elucidate the pathogenesis of chronic Lyme arthritis. Given the complexity of the phenomenon and the fact that causal mechanisms cannot be pursued in the murine model, one hopes that these new findings set this line of investigation on a steady course.

We thank the reviewer for his/her/their appreciation and feedback. We agree with all the points made.

Regarding the discrepancy, we agree and acknowledge that we can only offer possible explanations. We feel that a definitive explanation would be difficult to obtain as we no longer have the growth medium used in the 2019 study (preventing direct comparison). Importantly, our 2019 paper suggested two potential sources of immunogenic peptidoglycan fragments (one from live spirochetes and the other from dead ones), generating hypotheses that we tested in this follow-up study. We have revised the text to clarify these points.

Regarding the difficulty of elucidating the pathogenesis of chronic Lyme arthritis, we fully agree! It is a challenging but incredibly important endeavor. We believe that our study is an important step forward.

Reviewer #2: The manuscript by McCausland et al. reports on the interplay between Borrelia burgdorferi peptidoglycan and the immune response. The paper details that PG from dead Borrelia is more immunogenic than that extruded from live bacteria, identifies two key enzymes involved in PG turnover in the spirochete pathogen, and suggests that variation in a human PG degradation enzyme could explain both interpersonal and infection niche-level variation in disease outcomes. The study is well-designed and well-written (in fact, I truly enjoyed reading it), and yields impactful insight into the molecular mechanisms underlying Lyme disease progression. All controls are in place, and the conclusions are sound, and supported by the data. I only have very minor comments on the text.

We thank the reviewer for his/her/their time, kind comments, and helpful feedback.

Reviewer #3: The questions posed by the authors of this manuscript are significant in Bb immunopathogenesis, particularly the question of whether the immunogenic properties of Bb-PG are different against live vs dead organisms. The authors show that Bb MltS (BB0259) cleaves Bb-PG fragments, namely into 1,6-anhydromuramyl-contiaining fragments which are poor inducers of NOD2 immune responses. On the host side, the activity of PGLYRP2, an enzyme that cleaves Bb-PG to render it less immunogenic, is lower in joint fluids compared to serum, a finding that is consistent with previous clinical findings of Bb-PG-induced joint arthritis.

Major concerns focus on the conclusions drawn from the human patient samples and Bb-PG produced by “dead” vs. “live” Bb. Overall, this manuscript contains well-controlled experiments that would be helpful to those studying Bb immunopathogenesis from both host and bacteria perspectives.

We thank the reviewer for his/her/their time, appreciation, and valuable feedback. We address the major concerns below.

**Part II – Major Issues: Key Experiments Required for Acceptance**
Please use this section to detail the key new experiments or modifications of existing experiments that should be absolutely required to validate study conclusions.

Generally, there should be no more than 3 such required experiments or major modifications for a "Major Revision" recommendation. If more than 3 experiments are necessary to validate the study conclusions, then you are encouraged to recommend "Reject".

Reviewer #1: No major issues.

Thank you!

Reviewer #2: none

Thank you!

Reviewer #3:

The scientific question of comparing Bb-PG from live vs dead Bb is of great interest. While the authors cite data from Li et al. 2011 (discussed in lines 448-451) which demonstrates that Bb cannot be cultured in the presence of Lyme arthritis joint fluid, this manuscript exclusively analyzes Bb-PG produced by live Bb. The authors do a good job qualifying this correlation by using “likely dead”; however, they might consider experiments that could provide direct evidence. Some ideas include “starving” Bb of nutrients in BSK (ie. growing Bb until cultures become yellow and cells begin to die) or PBS-inactivation of Bb, using this as the input for the pipeline described in Fig 1B.

We have addressed this concern through textual revision and by adding experimental results. Previously, we showed that peptidoglycan harvested from cultures reaching saturation in cell density is pro-inflammatory (i.e., activates NOD2 and stimulates the production and secretion of cytokines and chemokines by human primary peripheral blood mononuclear cells) (Jutras et al, 2019). This peptidoglycan primarily contains the pro-inflammatory motif (MurNAc-L-Ala-D-Glu) recognized by NOD2, with the anhydromuramyl form being a minor component (Jutras et al, 2019). Following the reviewer’s suggestion, we have now harvested *B. burgdorferi* peptidoglycan from 10-day-old cultures in which over 90% of cells are known to be dead (i.e., are unable to form colonies upon nutrient repletion), as recently demonstrated (PMID 39950812). We found that the isolated peptidoglycan sacculi from these (dead) cells is still rich in pro-inflammatory MurNAc-containing monomer. These new experimental results are presented in the new Figure S13. The text has been revised accordingly.

Is it possible to characterize Bb-PG directly from the host joint fluid (either murine or human)? While the experiments put forth in this manuscript provide thorough mechanistic data of Bb-PG processing in vitro, it is widely understood that the transcriptional profile (which may affect the varieties of PG produced) is different between in vitro BSK culture and in vivo (most famously expression of OspA vs OspC). Have the authors tried performing LC/MS on joint fluid samples from Lyme arthritis patients (or infected/cured mice)?

Yes, we have tried, but our results were not conclusive because of the large technical challenges associated with this experiment. First, for such experiments, we need quite large volumes of joint fluid, which is difficult to obtain from patients and impossible from infected mice. Second, this is not a traditional mass spectrometry experiment in which one searches for a specific molecule that has a given mass in a complex fluid. The peptidoglycan material of dead spirochetes, particularly the one expected to persist in synovial tissues, is in multimeric/polymeric form, as suggested by our study and recently confirmed by a study published a few weeks ago (PMID: 40267217). Our current method can only detect peptidoglycan monomers and dimers, which is appropriate for shed PG material from live *Borrelia* cells (anything bigger cannot cross the outer membrane) but not for multimeric PG material for which the mass is unknown. For PG monomers and dimers alone, the computational possibilities of masses already exceed 700. Anything larger than a dimer gives rise to too many possible masses to be reliable. Currently, the best available method to detect polymeric *B. burgdorferi* peptidoglycan is with specific antibodies. We have previously provided evidence of peptidoglycan detection in the joint fluids of Lyme arthritis patients using such an antibody-based assay (Jutras et al, 2019). This result has been confirmed recently using an antibody-based assay specific to polymeric *Borrelia* peptidoglycan (PMID: 40267217). In addition, this recent study shows that unlike monomers, the polymeric form of *B. burgdorferi* peptidoglycan injected in mice persists in their bodies for weeks, consistent with our results and interpretations. The text has been revised to discuss these points.

The variability of human samples has been discussed heavily in the manuscript, but a few points stand out:

Bimodal data. For the serum samples in Fig 6B, the green and brown patients versus the red and orange patients cluster in almost a bimodal fashion. However, based on the data presented in Fig 6C, it seems that the distribution of PGLYRP2 ng/mL is evenly distributed. Does this suggest that other enzymes/factors may be at play?

We agree that there are likely other PG-degrading enzymes or relevant factors in the patient samples. However, this remains speculative as we cannot rule out that the differences in distributions are due to noise and spontaneous fragmentation associated with mass spectrometry. We also think that the linear scale of the Y-axis in the plot of Fig. 6B might have been confusing. Therefore, the data (which include additional results, see below for details) are now provided on both logarithmic and linear scales in Fig. 6B and Fig. S14B, respectively.

Another relevant point is that Fig. 6B shows the data only for a subset of the enzymatic products shown in Fig. 6A (complete set) as illustration. Therefore, Fig. 6C should be compared with the full peptidoglycan digest profile shown in Fig. 6A. They show trends across individuals that are largely (though not perfectly) consistent with the ELISA results (Fig. 6C). We have revised the text to make these points, including the suggestion of potential unidentified peptidoglycan-hydrolytic enzymes in the samples, as pointed out by the reviewer. Thank you for the suggestion.

Omission of blue patient. The joint fluid of the blue patient is not displayed in 6B, JF columns in the top row of panels. This datapoint is of particular interest, given the JF PGLYRP2 level relative to the PGLYRP2 level of the red patient in serum (Fig. 6C). If PGLYRP2 is responsible for amidase cleavage, one would expect that the extracted ion counts of the blue JF patient would match the peptide pattern of the red patient in the serum.

Thanks for bringing this to our attention. This was a plotting issue; the blue patient data was hidden by the other data points. In the revised manuscript, we also provide the same data in logarithmic scale (revised Fig. 6B) and linear scale (new Fig. S14B) to help with data visualization. In the log scale plot, the PGLYRP2-like activity in the joint fluid of the blue patient is not quite as high as in the serum of the purple (formerly red patient), but it is higher than in the joint fluids of other Lyme disease patients. So, the trends are conserved. It is certainly possible that there is a PGLYRP2-independent amidase activity in some samples, which we now mention in the revised text.

Healthy Controls. Measuring PGLYRP2 and Lysozyme (Fig. 6C) levels in JF and serum of healthy controls would be interesting. Could it be possible that the endogenous levels of these enzymes are lower/higher in Lyme patients due to inflammation/infection? One could imagine adding healthy control samples to 6A-C.

For the serum, we have added mass spectrometry and ELISA data (revised Fig. 6A-B and C, respectively) for a “healthy” control, which is a commercially available, pooled serum sample from many healthy individuals.

Unfortunately, joint fluids from healthy individuals are harder to come by. People tend to be unwilling to undergo the procedure of joint fluid extraction without a medical reason, and more importantly, there is little to no joint fluid to extract in the absence of inflammation. To circumvent this problem, we analyzed the joint fluids of three rheumatoid arthritis patients. These data are included in the revised Fig. 6 and associated supplementary Fig. S14.

**Part III – Minor Issues: Editorial and Data Presentation Modifications**
Please use this section for editorial suggestions as well as relatively minor modifications of existing data that would enhance clarity.

Reviewer #1: Results:
Lines 157-158. Since the mass spec data were matched against a computational library, it isn't clear how the authors could be certain that the PG fragments had an anhydro modification.

Our computational library includes the anhydro modification among its >750 permutations. This modification results in a mass difference of ~18 daltons (i.e., subtraction of a water molecule to account for the anhydro bond formation), and can therefore be detected by QTOF LC-MS. For detection, we use an accuracy cutoff of 20 ppm (see Materials and Methods), which allows us to confidently differentiate between anhydro and non-anhydro fragments. For example, for the predicted 850-Da mass of the predominant monomer Glc*N*Ac-AnhMur*N*Ac-l-Ala-d-Glu-l-Orn(Gly), 20 ppm corresponds to 0.017 Da. Furthermore, our tandem-mass spectrometry fragmentation profiles are also consistent with the presence of an anhydro bond (Fig. S4). Finally, the anhydromuramyl products are absent in the culture supernatants of the strain lacking the lytic transglycosylase MltS (Fig. 4) and transglycosylases are well known to produce PG fragments with anhydro bonds (PMIDS 9642199, 357, 28644060, https://doi.org/10.1111/j.1432-1033.1975.tb04040.x). We have revised the text to clarify these points.

Lines 241-244. Even after multiple readings, this reviewer finds this complex sentence difficult to understand.

We apologize for the confusion. The text has been revised to clarify its meaning.

Figure 6 and related text in Results. The authors should indicate that their data do not enable them to state definitively that the fragments observed are due to the specific enzymes mentioned.

We agree that Fig. 6A only informs us about the type of enzymatic activities in the samples and not about the specific enzymes, unlike Fig. 6B, which shows the levels of specific proteins. We have revised the text to speak activities and not specific proteins when describing the data in Fig. 6A.

Discussion:
-Most of the Discussion on p17 seems superfluous.

We agree that this section is speculative, but we clearly state that it is speculative, which seems reasonable for a discussion section. If possible, we would prefer to keep this section because it answers a question (why does *B. burgdorferi* shed PG fragments when it seems wasteful?) that we get almost every time that we present this work to a scientific audience.

-Readers would benefit if the current data were explained in a more straightforward fashion in the context of the author's previous PNAS 2019 paper. In other words, which results from the prior study are they building upon in the current work?

We modified the introduction to clarify how our previous work generated questions that we specifically address in this paper.

-Similarly, the innate and autoimmune mechanisms that presumably collaborate to cause Lyme arthritis could be presented as a model followed by supportive evidence.

Our paper connects innate immunity to Lyme arthritis but we do not provide direct evidence of a link to autoimmunity. Therefore, we feel that the presentation of a model linking the two would be premature. However, we indeed find the hypothesis of peptidoglycan-induced innate immunity contributing to autoimmunity very attractive (which we are investigating with collaborators).

Reviewer #2:

- Line 207, compared not compares
- Line 255 “subunits”
- Line 238 “increased”
- Line 242 “environment is derived”
- Line 292 “to be poor inducers”
- Line 299 “refer to PG fragments” (also delete “to” later in the same sentence

All typos were corrected. Thank you.

- Section starting with line 353 – the variability across patients is intriguing. How did you ensure that sampling and sample handling was consistent between all patients to exclude sampling artifacts as a source of variation? The description in the methods does state that samples were treated equally, but I wonder if those samples could be normalized to e.g. total protein content to make sure they were not inadvertently diluted during sampling? Alternative to normalizing, more information on the sampling in the Methods section would help (currently just states that samples were “collected”, but unclear how exactly”

We have expanded the Materials and Methods section to elaborate on our sample collection.

- Line 444 – this is a bit unclear. Since B. burgdorferi can scavenge PG from the environment, it must possess an uptake system and the cytosolic mechanisms to turn PG into a form it can use. How is this different from a canonical recycling pathway, unless the transporter somehow specifically recognizes and transports non-Borrelia PG?

The canonical PG recycling pathway transports PG fragments that must have an anhydro-*N*-acetylmuramyl residue to be recognized by the AmpG transporter (PMID: 12426329). *Borrelia* uses the chitobiose transporter ChbC to import extracellular chitin (polymeric or monomeric Glc*N*Ac) and digest it into monomers using the chitinases BB0002 and BB0620 (PMID: 20102636 and 34819646). We have added this information to the revised text.

Reviewer #3:

• Consistency of naming the control condition in Fig. 1 should be noted: in 1E and throughout the figure 1 legend, the control is referred to as “BSK”, while 1B refers to the control as “Medium only”. Consider changing 1B “BSK control”.

Good suggestion, thank you. The naming is now defined and consistent.

• While the Bb strains used in 2A are all infectious, did the authors perform any plasmid typing confirmation to ensure that these strains possess their full plasmid profile (and therefore carry the entire genome to produce the full spectrum of Bb-PG)?

To address the reviewer’s question, we performed whole-genome sequencing on all strains shown in Figure 2A. The results are presented in a new supplementary figure (Fig. S6). They show that three of them (B31 IR, K2, and N40) retain their full complement of plasmids. The fourth strain (297) is missing cp32-6 and cp32-9. However, a recent article (PMID 40108404) has shown that *B. burgdorferi* remains infectious without cp32 plasmids. Therefore, we expect all four strains to be infectious. The text has been revised to incorporate this new information.

• The statement made in line 381-383 (The results indicate that while both types of fluid display lysozyme activity...) may be misleading. While 6C does indeed show lysozyme presence (ug/mL, detected by ELISA) in both joint fluid and serum, it seems like activity is absent in serum, given that the extracted ion counts for Bb-PG products produced by lysozyme cleavage (bottom four panels of 6B) show little to no lysozyme cleavage above background levels.

We apologize for the confusion. The disaccharide-penta(gly) and -tri(gly) shown in Fig 6B are indeed lysozyme products. However, the reason they don’t accumulate in sera is likely because there is also N-acetyl-muramyl-L-Ala amidase (PGLYRP2-like) activity in sera that further degrades these peptidoglycan fragments into free disaccharides and peptides. Fig. 6C shows illustrative examples of PG fragments (which we now clarify in the revised text). The complete set is shown in Fig. 6A where the accumulation of disaccharides (“X” in the figure) can be seen in sera but not joint fluids. These disaccharides require a muramidase (lysozyme-like) activity to be released from the peptidoglycan sacculus. Their accumulation is consistent with the presence of lysozyme shown by ELISA (Fig. 6C). We have clarified this point in the text.
